# Genome-wide promoter methylation profile of human testis and epididymis: identified from cell-free seminal DNA

**DOI:** 10.1186/1471-2164-14-288

**Published:** 2013-04-28

**Authors:** Chunlin Wu, Xiaofang Ding, Honggang Li, Changhong Zhu, Chengliang Xiong

**Affiliations:** 1 Family Planning Research Institute/Center of Reproductive Medicine, Tongji Medical College, Huazhong University of Science and Technology, Wuhan, 430030, China; 2 Centre of Reproductive Medicine, Union Hospital, Tongji Medical College, Huazhong University of Science and Technology, Wuhan, 430022, China; 3 Wuhan Tongji Reproductive Medicine Hospital, Wuhan, 430013, China

**Keywords:** DNA methylation, Cell-free seminal DNA, Testis, Epididymis, Vasectomy, Noninvasive diagnosis

## Abstract

**Background:**

DNA methylation analysis is useful for investigation of male fertility in mammals, whereas the reliance on tissues limits the research on human. We have previously found the presence of high concentration of cell-free seminal DNA (cfsDNA) in human semen. We proposed that some testis and epididymis-specific methylated promoters could be detected in human cfsDNA, and thus hold promise as noninvasive epigenetic biomarkers for male infertility, of which most cases are caused by defects in testicular sperm production or epididymal sperm maturation.

**Results:**

The ejaculate of successfully vasectomized men does not contain any secretion from testis and epididymis. Here we compared genome-wide promoter methylation profiles in cfsDNA between health donors and post-vasectomy men. Promoters of 367 testis and epididymis-specific hypomethylated genes and 134 hypermethylated genes were identified. Subsequent validation by Methyl-DNA immunoprecipitation and MethyLight analysis confirmed the result of promoter microarray. Gene Ontology analysis revealed many genes involved in male reproduction.

**Conclusion:**

We detected the testis and epididymis-specific methylated promoters in human cfsDNA, which may be used for noninvasive epigenetic biomarkers for the study and diagnosis of male infertility.

## Background

Cell-free nucleic acids, including DNA and RNA, exist ubiquitously as cell-free or being absorbed at the cell surface of living organisms [1]. They are found to be released via apoptotic or necrotic cells, and be actively secreted by living cells [1,2]. Cell-free DNA, as a type of cell-free nucleic acids, can be isolated from various human body fluids including blood plasma [3,4], urine [5], cerebrospinal fluid [6], bronchoalveolar lavage fluid [7], amniotic fluid [8], and seminal plasma [9,10]. The isolated cell-free DNA can be used to examine DNA integrity, microsatellite instability, loss of heterozygosity, mutations, polymorphisms, and DNA methylation [2]. In recent years, considerable attention has been paid to taking advantage of tissue or cell-specific DNA methylation markers in cell-free DNA, which represents one of the most promising approaches for detection and prognosis of cancers, and prenatal diagnosis [2,4,8].

Cell-free seminal DNA (cfsDNA) has been detected in human semen [10]. Our previous work has isolated cfsDNA and described its characteristics [9]. Given that human ejaculate is a mixture secreted from bilateral testes, epididymides, seminal vesicles, bulbourethral glands, and the prostate [11], cfsDNA should contain DNA epigenetic information of these organs. Moreover, these DNA epigenetic information should be undetectable in the blood, because DNA should not pass the physical barrier between the blood and the male reproduction system including blood-testis barrier and blood-epididymis barrier.

DNA epigenetic modifications are essential for spermatogenesis. Remethylation occurs from the prospermatogonia stage onwards and results in mature spermatozoa with an adequate DNA methylation pattern [12]. The methylation status of some genes is changed in some conditions that cause male infertility [13]. We presumed that the promoters of testis and epididymis-specific methylated genes in cfsDNA should be promising markers for male infertility, because most conditions with male infertility are disordered in spermatogenesis and sperm maturation [14,15], which occur in testis and epididymis, respectively. In addition, environmental chemicals may cause spermatogenic defects and sperm motility abnormality via epigenetic changes [16,17]. So we reasoned that the testis and epididymis-specific methylated promoters can also be used for revealing the epigenetic mechanism of male infertility by chemicals.

However, the epigenetic information of testis and epididymis in cfsDNA is still unclear. Although only 10% human ejaculate is secreted by testis and epididymis [11], we can still hope to detect DNA epigenetic information of testis and epididymis-specific methylated genes in semen. Firstly, germ cells undergo a high level of apoptosis and get into seminiferous tubule during spermatogenesis in testis, and the epithelium releases amounts of microvesicles into glandular lumen via apocrine secretion in epididymis. These characteristics may bring a large quantity of DNA from the testis and epididymis into the semen. Our previous study also indicated this possibility [9]. Secondly, many testis-specific methylated promoters were found in human testis [18-22].

The present study compared the genome-wide promoter methylation profiles of cfsDNA between healthy donors and vasectomized men. The differentially methylated promoters should be testis and epididymis-specific because the ejaculate of a successfully vasectomized man does not contain secretion from testis and epididymis. As a result, we determined for the first time the testis and epididymis-specific promoter methylation profile in cfsDNA, which may provide noninvasive epigenetic biomarkers for male infertility in humans.

## Results

### Quantity of cfsDNA in healthy subjects and vasectomized men

The ejaculate of a man with successfully post-vasectomy (PV) should not contain any secretion from the testis and epididymis. Successful vasectomy was confirmed by the absence of sperm in ejaculate and no detection of *DDX4* mRNA in cell-free seminal RNA by RT-PCR [23]. In order to find whether there are a large amount of DNA originating from testis and epididymis in seminal plasma, we measured the cfsDNA concentration of healthy donors with normozoospermia (Nor) and men with PV. The averaged cfsDNA concentration of Nor (n = 12) was 1.23 ± 0.32 μg/ml seminal plasma, with ranges from 0.72 to 1.73 μg/ml. In contrast, the averaged cfsDNA concentration of PV (n = 11) was 0.33 ± 0.09 μg/ml seminal plasma, with ranges from 0.22 to 0.48 μg /ml. The averaged cfsDNA concentration of Nor was about quadruple of PV, which suggests more than 70% cfsDNA originates from testis and epididymis.

### Genome-wide detection of the testis and epididymis-specific hypo- and hypermethylated promotors by Methyl-DNA immunoprecipitation (MeDIP) microarray

**Figure 1:**
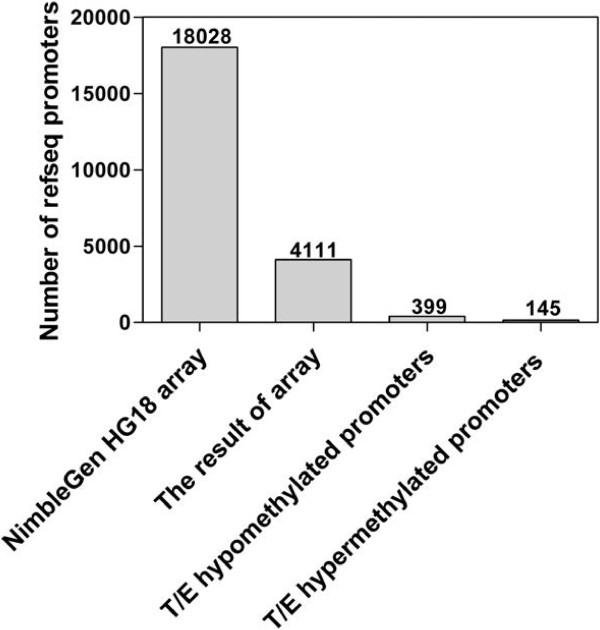
**Number of the testis and epididymis-specific hypo- and hypermethylated promoters in cfsDNA.** T/E refers to testis and epididymis-specific. The number on the top of each column denotes the quantity of promoters.

High concentration of cfsDNA in human semen and high proportion of cfsDNA from testis and epididymis make it possible to identify the epigenetic information of cfsDNA derived from testis and epididymis. We examined the methylation profiles of 18,028 human promoters in the seminal plasma of Nor and PV with the NimbleGem HG18 Refseq promoter array. Finally, we identified 4111 promoters in the cfsDNA of Nor and PV. Comparative analysis of the data of 4111 promoter methylation peaks in Nor and PV, we found 9.71% was testis and epididymis-specific hypomethylated promoters, and 3.53% was testis and epididymis-specific hypermethylated promoters (Figure 1). The multiple promoters from the same gene were summarized. Finally, we found 367 testis and epididymis-specific hypomethylated genes, and 134 testis and epididymis-specific hypermethylated genes. All genes are listed in Additional file 1.

**Figure 2:**
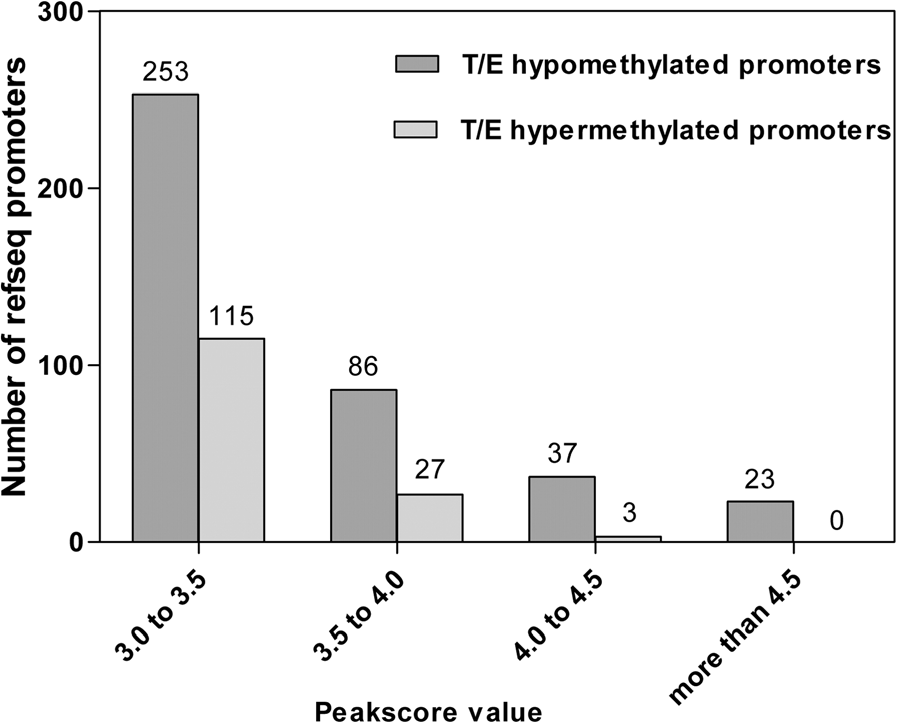
**The distribution of methylation peakscore value about the testis and epididymis-specific methylated promoters in cfsDNA.** The histogram indicates the distribution of testis and epididymis-specific hypo- and hypermethylated promoters according to the peakscore value, the number on the top of each column denotes the quantity of promoters in each range of peakscore value.

Considering the methylation peakscore value of promoters is associated with the probability of hypo- or hypermethylation, we summarized the distribution of peakscore value about the promoters of hypo- and hypermethylated genes so as to choose the reliable testis and epididymis-specific methylated promoters for the clinical research of male infertility. We found they showed the similar distribution (Figure 2). In the testis and epididymis-specific hypomethylated promoters, 63.41% of peakscore was between 3.0 and 3.5, and in the testis and epididymis-specific hypermethylated promoters, the percentage was 79.31%.

### Validation of the MeDIP microarray results

**Figure 3:**
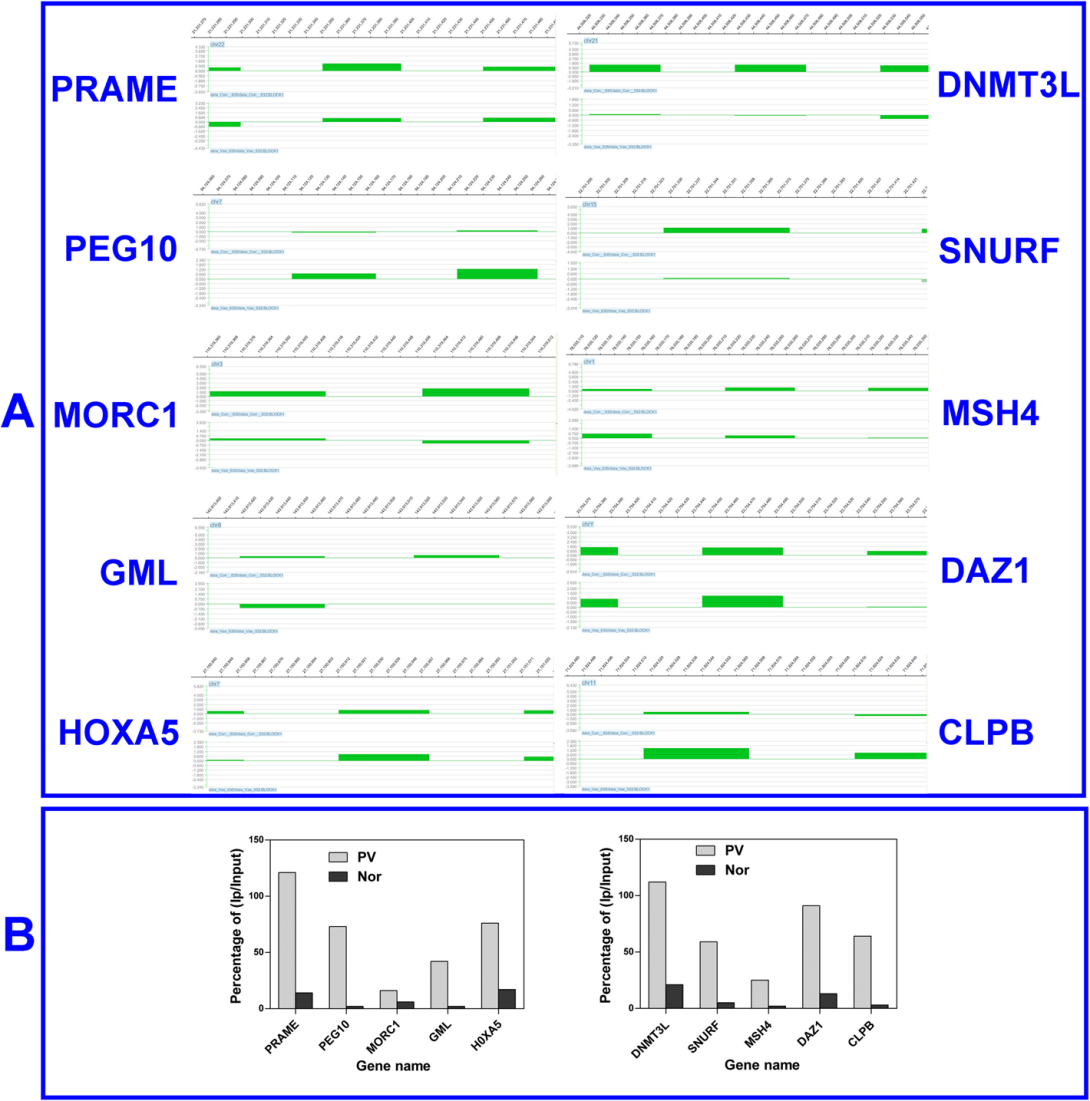
**Comparing the methylation status of 10 promoters between NimbleGen HG18 Promoter Array (A) and MeDIP-real time quantitative PCR (B). A**, The data of log_2_(IP/Input) graph shows the methylation signal intensity in the Promoter Array. The bars of upper row and lower row of lo_2_(IP/Input) graph in each amplifiable promoter fragments denote the methylation signal intensity of each probe in the cfsDNA of PV and Nor, respectively; **B**, MeDIP-real time quantitative PCR results. Each bar represents the methylation level of amplifiable promoter fragments analyzed by the relative quantity between IP and Input DNA.

To validate the dependability of MeDIP microarray data, the promoters of 10 testis and epididymis-specific methylated genes were analyzed by MeDIP-real time quantitative PCR, and of other 10 genes were analyzed by MethyLight. As shown in Figure 3, single-gene MeDIP result was concordant with the promoter methylation microarray, except for *PEG10* and *CLPB*. The methylation level of *GAPDH* was less than 0.0026% in Nor and less than 0.0045% in PV. The methylation level of *H19* was 59.30% in Nor and 43.47% in PV. MeDIP-real time quantitative PCR result is showed in Additional file 2. As for the other 10 promoters measured by MethyLight, nine promoters were successfully determined, except the failure of *MCM10* amplification. Eight testis and epididymis-specific methylated promoters were consistent with the promoter array besides *NCK2* (Additional file 3). In the detected 9 promoters, the difference of ΔCT between the unmethylated leukocyte DNA and fully methylated leukocyte DNA was all greater than 5.0. The result of MethyLight is showed in Additional file 4.

### The functions of testis and epididymis-specific hypo- and hypermethylated genes

Aiming to find the relationship between these testis and epididymis-specific methylated promoters and the biology of male reproduction, and suggest potential biomarkers for research and diagnosis of male infertility, Gene Ontology was used to analyze the biological processes and molecular functions of these genes. The most significant annotation clusters of enriched gene sets were inferred from functional annotation analysis with the testis and epididymis-specific hypo- or hypermethylated genes.

The biological processes of gene set analysis are shown in Tables 1 and 2. The testis and epididymis-specific hypomethylated genes were enriched for genes involved in sexual reproduction, ion transport, organic acid transport, negative regulation of transport, and defense response (Table 1). In contrast, the testis and epididymis specific hypermethylated genes were significantly related to regulation of cellular protein metabolic process, translation, cellular cation homeostasis, regulation of ubiquitin-protein ligase activity during mitotic cell cycle and regulation of epidermal growth factor receptor activity (Table 2).

**Table 1:** GO analysis of biological processes with testis and epididymis-specific hypomethylated genes

**Gene Ontology Term (GO:ID)**	**Number of genes**	**ES**^**a**^
Ion transport (GO:0006811)	21	1.19
Phosphorylation (GO:0016310)	21	1.05
Cell adhesion (GO:0007155)	20	1.28
Defense response (GO:0006952)	18	1.26
Protein complex biogenesis (GO:0070271)	15	1.12
Sexual reproduction (GO:0019953)	14	1.14
Ectoderm development (GO:0007398)	11	2.47
Nitrogen compound biosynthetic process (GO:0044271)	11	1.15
Sensory perception of light stimulus (GO:0050953)	9	1.37
Second-messenger-mediated signaling (GO:0019932)	9	1.20
Organic acid transport (GO:0015849)	7	1.28
Regulation of hormone levels (GO:0010817)	7	1.25
Muscle contraction (GO:0006936)	7	1.22
Negative regulation of transport (GO:0051051)	6	1.01

^a^ ES stands for Enrichment Score value of GO identifiers (ID), it equals (−log_10_P). *P* stands for the significance testing value of GO ID.

**Table 2:** GO analysis of biological processes with testis and epididymis-specific hypermethylated genes

**Gene Ontology Term (GO:ID)**	**Number of genes**	**ES**
Cell surface receptor linked signal transduction (GO:0007166)	20	1.51
Sensory perception of smell (GO:0007608)	9	2.13
Regulation of cellular protein metabolic process (GO:0032268)	8	1.44
Translation (GO:0006412)	6	1.17
Cellular cation homeostasis (GO:0030003)	5	1.06
Regulation of ubiquitin-protein ligase activity during mitotic cell cycle (GO:0051437)	3	1.10
Regulation of epidermal growth factor receptor activity (GO:0007176)	2	1.03

The molecular functions are shown in Additional file 5 and Additional file 6, the testis and epididymis-specific hypomethylated genes were enriched for genes relevant to structural molecule activity, channel activity, calcium ion binding, cytokine activity (Additional file 5); the hypermethylated genes were enriched for genes correlated to RNA binding and peptidase activity (Additional file 6).

## Discussion

The present study identified genome-wide testis and epididymis-specific promoter methylation information in cfsDNA, aiming to develop noninvasive epigenetic biomarkers for male infertility. For this purpose we compared promoter methylation profiles in the cfsDNA of Nor and PV. Overall, our results demonstrate that there are a number of promoters of testis and epididymis-specific hypo- and hypermethylated genes in cfsDNA, thus providing a foundation for developing noninvasive biomarkers for studying the epigenetic mechanism and clinical diagnosis of male infertility, of which most conditions are impaired testicular spermatogenesis and epididymal sperm maturation.

In our study, the semen samples of successful vasectomy are indispensable, because these samples do not contain DNA originating from testis and epididymis owing to the ligation of vas deferens, which makes it possible to screen the testis or epididymis-specific methylated promoters. Although several previous studies reported that vasectomy could change the mRNA and protein expression of epididymis and impair the spermatogenesis of testis and induce aberrant DNA methylation at imprinted genes in testicular sperm [24-27], there are no reports in the literature describing the change of DNA methylation status in human seminal vesicles, bulbourethral glands and the prostate after vasectomy. Therefore, comparative analysis of promoter methylation information in the cfsDNA of Nor and PV can identify variations in methylation profiles at promoter regions and thus identifies promoters that are likely testis and epididymis tissue specific and potential biomarkers for male fertility.

Another rationale for identification testis and epididymis-specific methylated promoters is the high contribution of releasing or secretion of these organs to cfsDNA concentration. We previously found that concentration of cfsDNA is much higher than other body fluids, and proposed the high contribution of testicular apoptosis to cfsDNA [9]. In the present study, averaged cfsDNA concentration in Nor was about quadruple of PV, indicating that cfsDNA is mostly originated from testis and epididymis. Considering spontaneous apoptosis happens in 75% of germ cell, which would develop to mature spermatozoa in mammalian, and the renewal of epididymal epithelium is slow [28,29], it is more likely that the apoptosis of germ cell in testis is the primary origin of cfsDNA from testis and epididymis.

Moreover, to ensure the reliability of testis and epididymis-specific methylated promoters identified by promoter methylation profile, we excluded some factors influencing the methylation profile of cfsDNA and kept the control subjects and vasectomized donors as matched as possible. Some previous studies reported that aberrant DNA methylation occurred in distinct diseases [30]. In addition, environmental chemicals [16,17], age [31] and lifestyle factors [32] can also affect the DNA methylation of human tissues. Therefore, to meet inclusion criteria, both Nor and PV groups were screened for potential confounding conditions such as inflammation of reproduction organs, genetic disease, potential occupational exposure, obesity and smoking and excess alcohol consumption.

By the microarray of promoter methylation, we identified the promoters of 367 testis and epididymis-specific hypomethylated genes and 134 testis and epididymis-specific hypermethylated genes. Comparing our result with other studies about DNA methylation information of human testis, we can determine some testis-specific methylated genes. Currently, only one study has globally analyzed the human testis-specific promoter methylation to identify testis-specific hypomethylated genes [18]. Totally 12 genes with promoter hypomethylation in this report are found in our results. Among them, *MORC1* is related to spermatogenesis [33]; *ELF5* is involved in cell proliferation [34]; *PRAME* is associated with the regulation of apoptosis [35]; *SPACA3* is correlated to the sperm-egg recognition [36]. But some testis-specific hypomethylated genes are inconsistent with our result. It may be mainly due to the differences in the comparison and source of DNA. The previous report [18] compared DNAs between testis and other tissues, whereas we compared cfsDNA between vasectomized men and healthy donors. In addition, the testicular DNA originates from spermatogenic cells, Sertoli cell and Leydig cell. However, testis and epididymis-specific cfsDNA should be mainly released by the apoptotic germ cells including spermatogonia, spermatocytes and spermatids [37], and epididymal epithelium. Some testis and epididymis-specific hypomethylated genes relevant to the spermatogenesis in our result, such as *DNMT3L, HSF1, MSH4, THEG, SOHLH1* and *CIB1*[38], cannot be found in the methylation profile of testis tissue owing to the interference of Sertoli cell and Leydig cell [18]. Some other reports studied promoter methylation of individual genes in testis [19,20]. Our result confirmed the testis-specific hypomethylated genes including *MAGEA1*[19] and *ANKRD30A*[20].

The result of single-gene MeDIP-real time quantitative PCR and MethyLight analysis confirmed the reliability of our microarray data. Among the 19 promoters which were successfully determined, sixteen are consistent with the result of promoter methylation microarray. The other 3 promoters tested as false positives on the microarray, which may be due to the possibly inefficient or non-specific detection of individual probes on the microarray and normalization artifacts during data processing. The failure of measurement of *MCM10* may be due to the inappropriate primers and probe. Several previous studies concurred with our result of single-gene MeDIP and MethyLight analysis: the hypomethylated promoters of *CLPB*, *PRAME, DAZ1 MORC1*, and *NCK2* were confirmed in human testis [18]; the hypomethylated promoters of *HOXA5, GML, PEG10* and *SNURF* were identified in human sperm [15,39]. Methylation level of *H19* in single-gene MeDIP analysis is lower than human spermatocytes and spermatid, but it is similar with spermatogonia and somatic cell [40], which suggests that most of *H19* in cfsDNA originates from the spermatogonia and epithelial cell of male reproduction organs.

To further understand the potential function of testis and epididymis-specific hypo- and hypermethylated genes, and apply these potential epigenetic markers, we carried out Gene Ontology analysis with the testis and epididymis-specific hypomethylated genes and hypermethylated genes, respectively. Out of the biological processes of testis and epididymis-specific hypomethylated genes, it is obviously that sexual reproduction is consistent with the function of testis because the process of spermatogenesis takes place in testis and involves the development of mitotically growing spermatogonia into meiotic spermatocytes that give rise to haploid spermatids, and subsequently differentiate into mature sperm [41]. Other biological processes, such as ion transport, organic acid transport and negative regulation of transport, participate in the reabsorption and excretion of epididymis [14], the biological process of defense response may protect the sperm from outside bacterial invasion in epididymis [42]. On the other hand, the biological processes of testis and epididymis-specific hypermethylated genes involve in regulation of cellular protein metabolic process, regulation of ubiquitin-protein ligase activity during mitotic cell cycle and regulation of epidermal growth factor receptor activity, which may be the apoptotic inducement of spermatogenic cell because of either low expression or silence of these hypermethylated genes.

## Conclusion

Taken together, we have firstly identified the genome-wide testis and epididymis-specific hypo- and hypermethylated promoters in cfsDNA. Totally 367 hypomethylated gene promoters and 134 hypermethylated gene promoters were proposed as testis and epididymis-specific, and can be detected in cfsDNA. The methylation modification of testis or epididymis-specific promoters is important for the spermatogenesis and sperm mature [12], and the aberrant methylation change of these promoters correlates with male infertility, carcinogenesis and development [12,16,17,43]. Therefore, the genome-wide testis or epididymis-specific methylated promoters in cfsDNA can be used as the potential epigenetic biomarkers for noninvasive study and diagnosis of male infertility, and the tumor of testis and epididymis.

## Methods

### Semen samples

All normozoospermic semen samples (n = 12) were collected from specimens remaining after routine andrological analysis. PV semen samples were acquired from adults (n = 11) who underwent vasectomy within 5 years. PV was confirmed by the absence of sperm in ejaculate and no detection of *DDX4* mRNA in cell-free seminal RNA by RT-PCR [23]. PV semen with any abnormal parameter other than sperm amounts was excluded. The semen criteria for Nor were according to guidelines of WHO (World Health Organization) [44]. The inclusion subjects for each group were 28-40 years old, the Han nationality, smoking <2 pack per year and intaking of alcohol <1 drink per week, body mass index between 18.5 and 24. All individuals had no family history of genetic diseases, history of autoimmune disorders, malignancy, hyperpyrexia, sexually transmitted diseases or inflammation of reproductive organs. Individuals who worked in high temperature environments or had occupational exposure to toxic chemicals or radiation were also excluded. This study was approved by the Institutional Review Board, and all participants provided written informed consent. Semen specimens were obtained by masturbation after 3-5 days of sexual abstinence and were allowed to liquefy for 30-60 min at room temperature.

### CfsDNA isolation and fragmentation

Seminal plasma was obtained by low speed centrifugation (400 × g for 10 min) to avoid cell lyses and then centrifuged again at 12, 000 × g for 10 min. The supernatant was carefully collected and subjected to cfsDNA extraction, which was performed as our previous described [9]. To obtain enough cfsDNA, 2.4 ml seminal plasma in every sample of 8 control subjects and 7 vasectomized men was abstracted. Concentration and purity of DNA were detected by an ultraviolet photometer (Biometra, Göttingen, Germany). DNA size distribution was detected using agarose gel electrophoresis. To reach the requirement of DNA quantity in promoter methylation microarray, and avoid individual variation, cfsDNA from 6 Nor and 6 PV were combined to make “Nor” and “PV” pools, respectively. Concentration of DNA was assessed by spectrophotometry for MeDIP. Ultrasonication to a mean fragment size of 200-500 bp was carried out with the bioruptor (Diagenode) using the following settings: Bioruptor on low, sonication for 10 cycles of 30 sec on and 30 sec off, and 5 μg DNA in 300 μl TE buffer. Fragment range was controlled using agarose gel electrophoresis.

### MeDIP and microarray hybridization

MeDIP was performed as previous described with some modification [45]. Briefly, 4 μg of fragmented DNA was used for a standard MeDIP assay. After denaturation at 95°C for 10 min, immunoprecipitation was performed using 4 μg mouse monoclonal antibody against 5-methylcytidine (1 μg/μl, Diagenode) in a final volume of 500 μl IP buffer (0.5% NP40, 1.1% Triton X-100, 1.5 mM EDTA, 50 mM Tris-HCl, 150 mM NaCl) at 4°C for 12 h. Immunoprecipitated complexes were collected with magnetic beads (Bangs laboratories. Inc) coupled anti-mouse IgG (1 μg/μl, Jackson) at 4°C for 2 h, washed with IP buffer for five times. DNA was purified by phenol-chloroform extraction and ethanol precipitation.

The Input and IP DNA were labeled with cy3- and cy5-labeled random nonamers, respectively. Labeled DNA from the Input and IP pools was mixed (Input/IP =1:1, 2 μg) and hybridized to NimbleGem HG18 Refseq promoter array (Roche, Germany), which contained all known well-characterized 18028 RefSeq promoter regions [(from about -2200 bp to +500 bp of the transcription start sites] totally covered by 385, 000 probes. Arrays were then washed and scanned with Axon GenePix 4000B microarray scanner. The raw data was abstracted as pair files by NimbleScan v2.5 (Roche-NimbleGen) software. After normalization, raw data was inputted into SignalMap software (v1.9, Roche-NimbleGen) to observe and evaluate the differential methylation peaks between Nor and PV cfsDNA.

A customized peak-finding algorithm supplied by NimbleGen was using to analyze methylation data from MeDIP-microarray (NimbleScan v2.5; Roche-Nimble-Gen) as previously described [46]. After scaled log_2_(IP/Input) data was generated, the modified algorithm was applied to carry out the one-sided Kolmogorov-Smirnov test on several adjacent probes using sliding windows to predict enriched regions across the microarray. The algorithm was set with specified parameters [sliding window width, 750 bp; minimal probes per peak, 2; *P* value (after -log_10_ transformation) minimum cutoff, 2; maximum spacing between nearby probes within peak, 500 bp] [47]. Then, the identified methylation peaks were mapped to genomic transcripts. Processed data was imported into microsoft office excel 2003 for further analysis. Promoters with significant methylation change were revealed according to the difference of methylation peaks in the cfsDNA of Nor and PV.

### Criteria for seminal promoters derived from testis and epididymis

The candidates of seminal promoters derived from testis and epididymis were selected based on the followong criteria: the peakscore value (-log_10_p) of testis and epididymis-specific hypomethylated promoters is more than 3.0 (*P* < 0.001) in the vasectomized men and less than 2.0 (*P* > 0.01) in the Nor donors; on the contrary, the peakscore value of testis and epididymis-specific hypermethylated promoters is more than 3.0 in the Nor donors and less than 2.0 in the vasectomized men. These promoters with obviously differential peakscore in Nor and PV are supposed to be released or secreted from testis and epididymis, because the ejaculate of a successfully vasectomized men does not contain secretion from testis and epididymis.

### Real-time quantitative PCR

Primers were designed using primer 5.0 and beacon designer 7.0 software and the quality was controlled by PCR and BLAT functions of the UCSC Genome Browser (http://genome.ucsc.edu/). Enrichment of a specific fragment in the MeDIP eluate was detected and quantified relative to the Input DNA by real-time quantitative PCR on an Mx3000P thermocycler (Stratagene) using the SYBR® Premix Ex Taq™ II (TaKaRa Code: DRR820A) according to the manufacturer’s description. We used *GAPDH* as the negative control and *H19* as the positive control to certificate the specificity of MeDIP because *GAPDH* promoter is normally unmethylated and *H19* promoter is normally 100% methylation in spermatid and 50% methylation in somatic cells [40]. Primer sequences were given in Additional file 7 and the positional relationship of primer, methylation peak and transcript start site in 10 genes selected for validation was shown in Table 3. Cycling conditions were as follows: denaturation (95°C for 30 sec), amplification and quantification (95°C for 5 sec, 58°C for 20 sec, 72°C for 20 sec with a single fluorescence measurement at the end of segment) repeated 40 times, a melting curve program (60-95°C with a heating rate of 0.1°C/sec and continuous fluorescence measurement) and a cooling step to 4°C.

**Table 3:** The positional relationship of primer, methylation peak and transcript start site (TSS) in genes selected for validation

**Gene**	**Gene name**	**GeneBank accession no.**	**Primer location(5′-3′)**		**Peak location**	**Strand**	**Position(TSS = +1)**	
			**Forword**	**Reverse**			**Forword 5′**	**Reverse 5′**
***PRAME***	Preferentially expressed antigen in melanoma	NM_206956	chr22:21231279	chr22:21231496	chr22:21231050	-	251	54
			−21231299	−21231479	−21231499			
***PEG10***	Paternally expressed 10	NM_015068	chr7:94124064	chr7:94124274	chr7:94123914	+	492	702
			−94124086	−94124252	−94124663			
***MORC1***	MORC family CW-type zinc finger 1	NM_014429	chr3:110319361	chr3:110319519	chr3:110319258	-	322	164
			−110319381	−110319495	−110320007			
***GML***	Glycosylphosphatidylinositol anchored molecule like protein	NM_002066	chr8:143913400	chr8:143913599	chr8:143912618	+	182	381
			−143913419	−143913577	−143913667			
***HOXA5***	Homeobox A5	NM_019102	chr7:27150841	chr7:27151029	chr7:27150312	-	−1029	−1217
			−27150862	−27151008	−27151961			
***DNMT3L***	DNA(cytosine-5-)- methyltransferase 3-like	NM_175867	chr21:44506322	chr21:44506560	chr21:44506027	-	205	−33
			−44506343	−44506540	−44508076			
***SNURF***	SNRPN upstream reading frame	NM_005678	chr15:22751295	chr15:22751430	chr15:22751327	+	68	203
			−22751314	−22751410	−22751676			
***MSH4***	MutS homolog 4	NM_002440	chr1:76035117	chr1:76035360	chr1:76035117	+	−100	143
			−76035135	−76035338	−76035566			
***DAZ1***	Deleted in azoospermia 1	NM_004081	chrY:23754371	chrY:23754582	chrY:23754045	-	256	45
			−23754389	−23754560	−23754994			
***CLPB***	Caseinolytic peptidase B homolog	NM_030813	chr11:71824486	chr11:71824650	chr11:71823916	-	−1270	−1434
			−71824504	−71824631	−71824774			

The methylation level of selected promoters in cfsDNA is calculated according to the relative quantity of MeDIP DNA to Input DNA in amplified fragments. Computing the relative quantity of each MeDIP DNA fraction to Input DNA requires to account for the difference of concentration in prepared DNA samples, so the percentage of IP/Input = 2^-[Ct(IP)–Ct(Input)]^ × (dilution factor of Input DNA) × 100% (Ct, cycle threshold). The size of products was identified by electrophoretically separated on 2.0% agarose gels.

### Bisulfite treatment of cfsDNA and MethyLight analysis

The seminal cfsDNA of 4 Nor and 4 PV was recovered as our previous described [9]. Sodium bisulfite conversion of cfsDNA was performed using EpiTect Bisulfite (Qiagen, Hilden, Germany). Following sodium bisulfite conversion, cfsDNA was analyzed using the MethyLight technique [48]. The primers and probe for *β-ACTIN* were previously reported [49], which served as a reference to normalize the quantity of input DNA. The other 10 sets of primers and probes, which were designed specifically by beacon designer 7.0 for bisulfite-converted cfsDNA, were used to detect the methylation level of testis and epididymis-specific hypo- or hypermethylated promoters. The sequences of primers and probes are given in Additional file 8. Specificity of the reactions for methylated DNA was confirmed separately using M.SssI-treated human leukocyte DNA (NEB, M0226L, USA) and unmethylated leukocyte DNA. TaqMan PCRs with primers and probe specific for the bisulfite-converted methylated promoter sequence of a particular locus and with the *β-ACTIN* reference primers and probe were performed separately. The percentage of methylation at a specific locus is to be 2^-ΔΔCt^ × 100%, where ΔΔCT = (CT_Target_-CT_Reference_)sample-(CT_Target_ -CT_Reference_)fully methylated DNA [50].

### Annotation of the testis and epididymis-specific hypo- and hypermethylated genes

For exploring potential shared functional of testis and epididymis-specific hypo- and hypermethylated genes, we used Gene Ontology (http://david.abcc.ncifcrf.gov/summary.jsp) from Functional Annotation Tools (http://david.abcc.ncifcrf.gov/tools.jsp) for Gene Ontology analysis and identified the statistical significance of the overlap with each Gene Ontology functional category using the Fisher exact test. Benjamini-Hochberg multiple testing correction was used to control false discovery rate, and the genes were classified into three main categories: cellular component, biological process, and molecular function.

## Abbreviations

cfsDNA: Cell-free seminal DNA; MeDIP: Methyl-DNA immunoprecipitation; PV: Post-vasectomy; Nor: Normozoospermia.

## Competing interests

The authors declare that they have no competing interests.

## Authors’ contributions

HL and XD elaborated the experimental design. CW and HL performed the microarray experiments and confirmed the reliability of microarray result. CW analyzed data, XD and CZ did the statistical analysis. CW wrote the draft. HL, CZ, and CX revised the manuscript. All authors discussed the results and commented on the manuscript. CX supervised the study. All authors read and approved the final manuscript.

## Supplementary Material

Additional file 1 The lists of testis and epididymis-specific hypo- and hypermethylated genes. Click here for file

Additional file 2 The Ct value of 10 validated genes in IP and Input DNA identified by MeDIP-real time quantitative PCR and the relative quantity of IP/Input in the cfsDNA of Nor and PV. Click here for file

Additional file 3 **The methylation status of 9 promoters analyzed by MethyLight.** Box plots illustrating the methylation level of amplifiable promoter fragments measured by MethyLight between the bisulfite-converted cfsDNA of 4 Nor and 4 PV. Methylation targets are promoter sequences specific to the 3 testis and epididymis-specific hypermethylated genes (*CHRFAM7A, USP28* and *NCK2*) and 6 testis and epididymis-specific hypomethylated genes (*ERRFI1, FBRS, GLTPD1, HSF1, VPS16* and *ZNF623*) identified by promoter methylation microarray. The boxes represent the quartiles and whiskers mark the range of the data, the horizontal line in the boxes denotes the median. Click here for file

Additional file 4 The Ct value of 9 validated genes identified by MethyLight analysis and the relative methylation level of target genes in the cfsDNA of Nor and PV. Click here for file

Additional file 5 **GO enrichment analysis of the testis and epididymis-specific hypomethylated genes.** Each bar represents the enrichment score (ES) of significant GO terms. The ES of each GO terms is larger than 1.0. The number in brackets denotes the quantity of genes involved in each GO term. Click here for file

Additional file 6 **GO enrichment analysis of the testis and epididymis-specific hypermethylated genes.** Each bar represents the enrichment score (ES) of significant GO terms. The ES of each GO terms is larger than 1.0. The number in brackets denotes the quantity of genes involved in each GO term. Click here for file

Additional file 7 Primers used for MeDIP-real time quantitative PCR. Click here for file

Additional file 8 Primers and probes used for MethyLight analysis. Click here for file
