# Supplementary figures and images for: Genome-wide promoter methylation profile of human testis and epididymis: identified from cell-free seminal DNA
Source: BMC Genomics. 2013 Apr 28;14:288. doi: 10.1186/1471-2164-14-288 (PMC3653781; doi:10.1186/1471-2164-14-288)

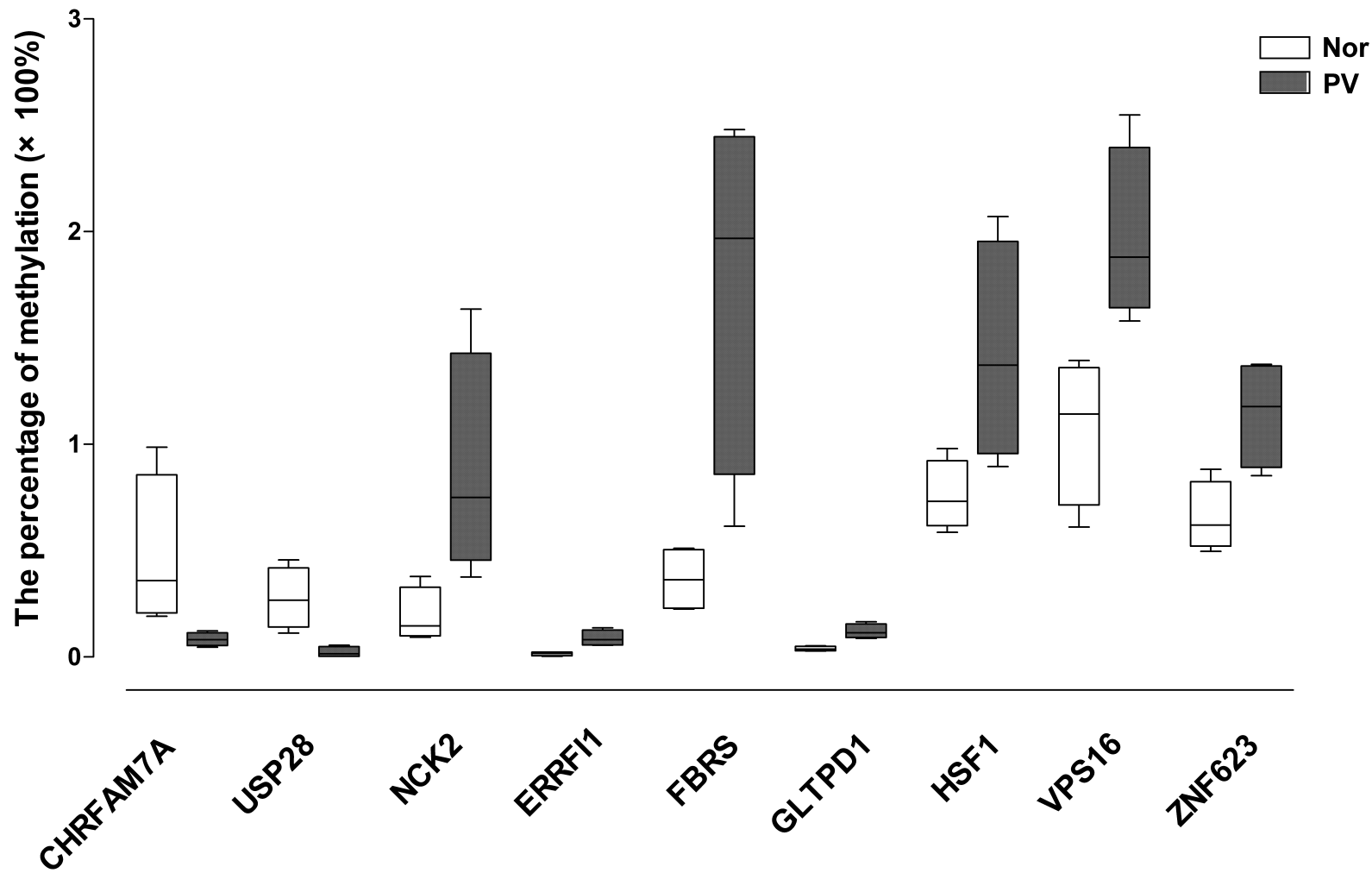

Supplement: Additional file 3 — The methylation status of 9 promoters analyzed by MethyLight. Box plots illustrating the methylation level of amplifiable promoter fragments measured by MethyLight between the bisulfite-converted cfsDNA of 4 Nor and 4 PV. Methylation targets are promoter sequences specific to the 3 testis and epididymis-specific hypermethylated genes (CHRFAM7A, USP28 and NCK2) and 6 testis and epididymis-specific hypomethylated genes (ERRFI1, FBRS, GLTPD1, HSF1, VPS16 and ZNF623) identified by promoter methylation microarray. The boxes represent the quartiles and whiskers mark the range of the data, the horizontal line in the boxes denotes the median. [file 1471-2164-14-288-S3.pdf]

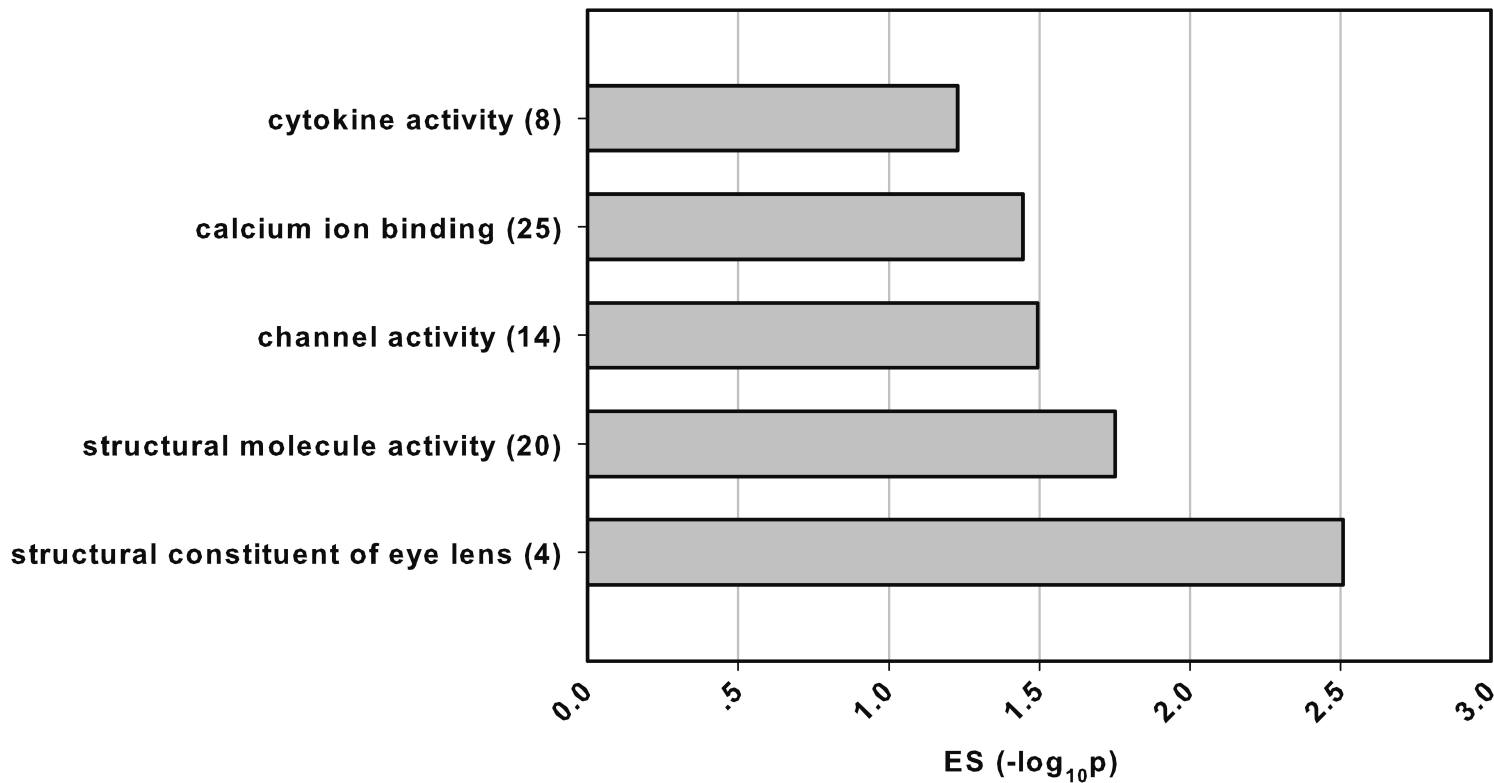

Supplement: Additional file 5 — GO enrichment analysis of the testis and epididymis-specific hypomethylated genes. Each bar represents the enrichment score (ES) of significant GO terms. The ES of each GO terms is larger than 1.0. The number in brackets denotes the quantity of genes involved in each GO term. [file 1471-2164-14-288-S5.pdf]

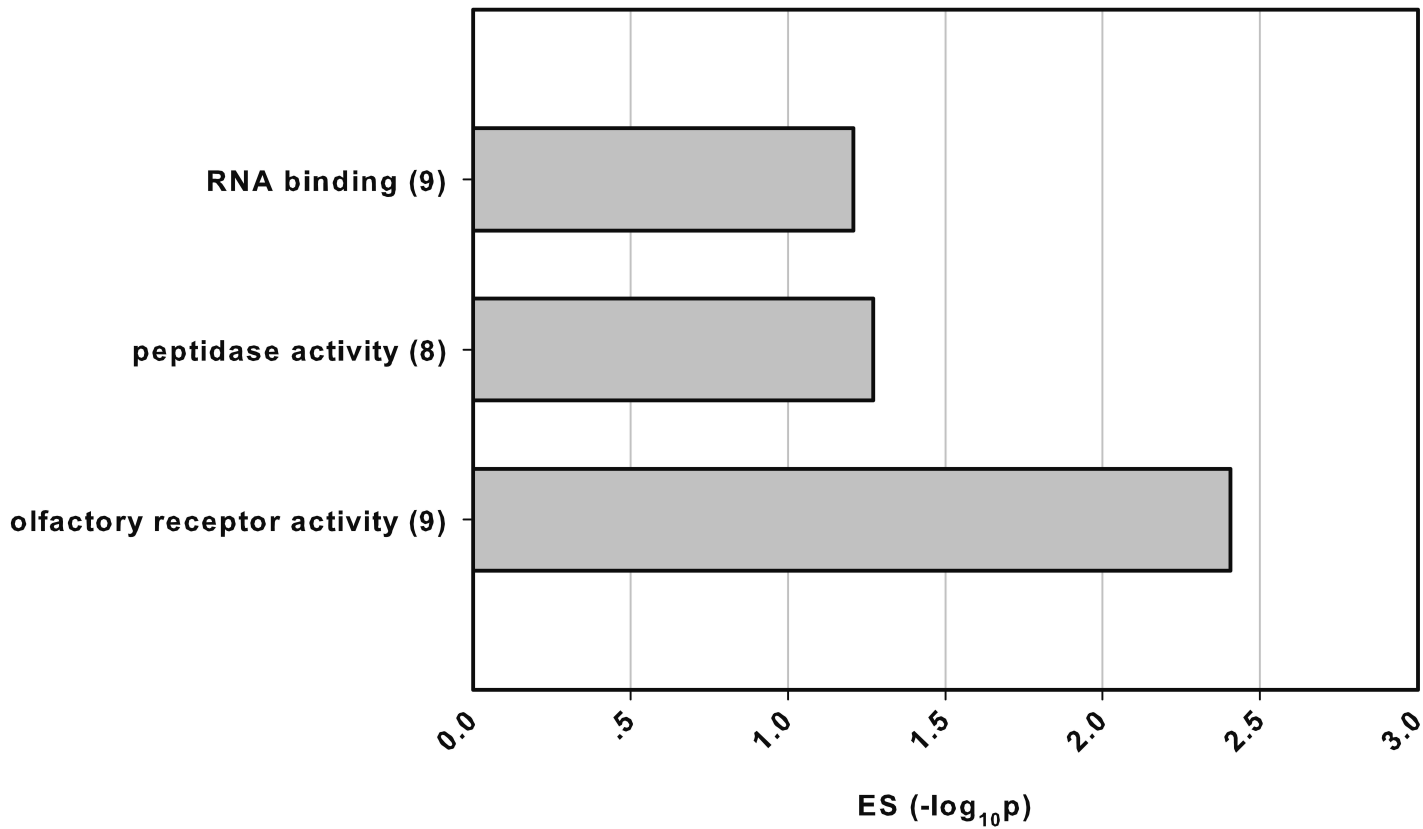

Supplement: Additional file 6 — GO enrichment analysis of the testis and epididymis-specific hypermethylated genes. Each bar represents the enrichment score (ES) of significant GO terms. The ES of each GO terms is larger than 1.0. The number in brackets denotes the quantity of genes involved in each GO term. [file 1471-2164-14-288-S6.pdf]
